# Does Osmotic Stress Affect Natural Product Expression in Fungi?

**DOI:** 10.3390/md15080254

**Published:** 2017-08-13

**Authors:** David Overy, Hebelin Correa, Catherine Roullier, Wei-Chiung Chi, Ka-Lai Pang, Mostafa Rateb, Rainer Ebel, Zhuo Shang, Rob Capon, Gerald Bills, Russell Kerr

**Affiliations:** 1 Department of Chemistry, University of Prince Edward Island, 550 University Avenue, Charlottetown, PE C1A 4P3, Canada; david.overy@canada.ca (D.O.); hcorreav@gmail.com (H.C.); 2 Department of Pathology and Microbiology, Atlantic Veterinary College, University of PEI, 550 University Avenue, Charlottetown, PE C1A 4P3, Canada; 3 Nautilus Biosciences Canada Inc., 550 University Avenue, Charlottetown, PE C1A 4P3, Canada; 4 Mer Molécules Santé—EA 2160, UFR des Sciences Pharmaceutiques et Biologiques, Université de Nantes, 9 Rue Bias, 44035 Nantes, France; catherine.roullier@univ-nantes.fr; 5 Department of Food Science, National Quemoy University, Kinmen County 89250, Taiwan; joan@nqu.edu.tw; 6 Institute of Marine Biology and Centre of Excellence for the Oceans, National Taiwan Ocean University, 2 Pei-Ning Road, Keelung 20224, Taiwan; klpang@mail.ntou.edu.tw; 7 Marine Biodiscovery Centre, Department of Chemistry, University of Aberdeen, Meston Walk, Aberdeen AB24 3UE, Scotland, UK; m.rateb@abdn.ac.uk (M.R.); r.ebel@abdn.ac.uk (R.E.); 8 School of Science & Sport, University of the West of Scotland, Paisley PA1 2BE, UK; 9 Division of Chemistry and Structural Biology, Institute for Molecular Bioscience, The University of Queensland, 306 Carmody Road, St. Lucia QLD 4072, Australia; z3shang@ucsd.edu (Z.S.); r.capon@uq.edu.au (R.C.); 10 Texas Therapeutics Institute, The Brown Foundation Institute of Molecular Medicine, University of Texas Health Science Center, 1881 East Rd., Houston, TX 77054, USA; Gerald.F.Bills@uth.tmc.edu; 11 Department of Biomedical Sciences, Atlantic Veterinary College, University of Prince Edward Island, 550 University Avenue, Charlottetown, PE C1A 4P3, Canada

**Keywords:** fungi, metabolite expression, LC-MS, metabolome, osmotic stress

## Abstract

The discovery of new natural products from fungi isolated from the marine environment has increased dramatically over the last few decades, leading to the identification of over 1000 new metabolites. However, most of the reported marine-derived species appear to be terrestrial in origin yet at the same time, facultatively halo- or osmotolerant. An unanswered question regarding the apparent chemical productivity of marine-derived fungi is whether the common practice of fermenting strains in seawater contributes to enhanced secondary metabolism? To answer this question, a terrestrial isolate of *Aspergillus aculeatus* was fermented in osmotic and saline stress conditions in parallel across multiple sites. The ex-type strain of *A. aculeatus* was obtained from three different culture collections. Site-to-site variations in metabolite expression were observed, suggesting that subculturing of the same strain and subtle variations in experimental protocols can have pronounced effects upon metabolite expression. Replicated experiments at individual sites indicated that secondary metabolite production was divergent between osmotic and saline treatments. Titers of some metabolites increased or decreased in response to increasing osmolite (salt or glycerol) concentrations. Furthermore, in some cases, the expression of some secondary metabolites in relation to osmotic and saline stress was attributed to specific sources of the ex-type strains.

## 1. Introduction

Genome sequencing confirms that fungi possess many more secondary metabolite gene clusters than are expressed in fermentations employing conventional methods and feedstocks. The impact of fermentation conditions and nutritional composition on secondary metabolite expression has been well documented and even minor variations in medium composition, dissolved oxygen, or temperature may modulate secondary metabolite biosynthesis [1,2]. Secondary metabolite-annotated gene clusters that remain transcriptionally silent even when strains are grown on a wide variety of media have been termed as “orphan”, “silent”, or “cryptic”; many of these have the potential to encode for new natural products [3]. Transcriptomic studies have revealed that exposure to stress can lead to transcription of “orphan” gene clusters [4]. Gene regulation studies involving halophilic fungi indicate that, although individual fungi exhibit different strategies for osmoregulation [5], differentially expressed genes encoding ion and metabolite transporters were found to be upregulated as a shared response due to salt stress (halotolerance) [6,7,8]. A link between secondary metabolite production as a response to salt stress is evident from comparative studies where differential expression in secondary metabolite production was observed from a strain of *Aspergillus terreus* (isolated from a saltern) and a marine-derived strain of *Mariannaea elegans* (as *Spicaria elegans*) due to increasing salinity of the cultivation medium compared to water controls [9,10].

The last two decades have seen a rapid increase in the discovery of new natural products from fungi isolated from substrata originating in marine habitats. In such reports, the natural products are often referred to as being “marine-derived”. The majority of most frequently cited marine-derived taxa are associated with ruderal substratum relationships, belonging to well-known osmotolerant terrestrial genera, such as *Aspergillus* and *Penicillium* [11,12,13]. Inversely correlated with the increased attention given to chemistry of marine-derived fungi has been a remarkable decline in natural products exploration of terrestrial soil fungi, as they are unjustly perceived as an exhausted resource of new natural products. However, genomics studies clearly indicate this bias is unfounded [14]. These points raise the question as to whether the use of seawater and artificial seawater supplements in media formulations affects the perceived distinctiveness of the chemistry of marine-derived isolates relative to their terrestrial counterparts? Few authors describing new natural products from marine-derived fungi have questioned whether genotypically related strains of the same species from a terrestrial habitat might produce the same compounds under identical fermentation conditions [15,16].

In August 2014, a workshop held at the University of Prince Edward Island, Charlottetown, Canada brought together an international group of mycologists and marine fungal natural product chemists to discuss topics relevant to marine fungal natural product research. The MaFNaP (Marine Fungal Natural Product) consortium was consequently established and some of the initial members are co-authors of this report. The question of whether culturing a fungus in the presence of seawater has an effect upon the secondary metabolic expression of the organism was debated. We hypothesize that the addition of salt in the culture medium exerts a selective physiological bias on the metabolic output of the fungus, as stress regulatory systems and fungal development are interconnected and directly affect the regulation of secondary metabolism. We also used this as an opportunity to rigorously examine the reproducibility of the “same experiment” conducted by investigators following a defined protocol in different parts of the world. To these ends, experiments were carried out at six different sites, where ex-type strains of *Aspergillus aculeatus* (a tropical soil isolate) were purchased directly from different culture collections or shared by participating labs. The strains were cultured under a standardized set of osmotic stress conditions. The effect upon secondary metabolite output was compared using five different osmotic conditions: natural seawater equivalent to 50% and 100% seawater salinity, glycerol at a water potential equivalent to 50% and 100% seawater salinity, and a 0% osmolite control.

## 2. Materials and Methods

### 2.1. Strain and Medium Selection

The ex-type strain of *A. aculeatus* was either obtained from three different culture collections (NRRL 20623, ATCC 16872, BCRC 32190) or distributed among the seven participating laboratories. This strain was chosen because it was easily accessible in public collections, and the draft genome sequence of this strain was available at the Joint Genome Institute [17], and a bioinformatic analysis of its genome revealed an extraordinarily rich secondary metabolism [18]. A basal medium was selected for fermentation studies that was comprised of: mannitol (40 g), maltose (40 g), yeast autolysate (Sigma Aldrich 73145, 10 g), K_2_HPO_4_ (2 g), MgSO_4_.7H_2_O (0.5 g), FeSO_4_.7H_2_O (0.01 g) in 1 L of distilled water. The basal medium was then used to prepare five different formulations of osmotic and seawater-enriched media designated as: 100% natural seawater (filtered seawater obtained locally), 50% natural seawater (a 1:1 dilution using deionized water), 100% glycerol (55.2 g/L of glycerol; an equivalent osmotic potential to the average salinity of seawater), 50% glycerol (27.6 g/L of glycerol), and 0% salt/glycerol (no salt/no glycerol control; basal medium formulation only). All flasks containing media were autoclaved at 121 °C for 20 min and left to cool to room temperature prior to inoculation. At each site, natural seawater was collected at the nearest access to the ocean and was stored at 4 °C until used. Seed inoculum was generated for fermentation studies for the three ex-type strains using the basal medium, incubated at 22 °C, shaking at 200 rpm for 5 days. 

### 2.2. Multi-Site Study

For the multi-site study, a single ex-type strain subculture was used per site for fermentation studies. The ex-type strain NRRL 20623 was distributed between the sites located in Nantes (France), Aberdeen (Scotland), Charlottetown (PE, Canada), and Houston (TX, USA); while the ex-type strain ATCC 16872 was obtained by the group in Brisbane (Australia), and the ex-type strain BCRC 32190 was obtained from the Bioresource Collection and Research Center by the group in Keelung (Taiwan). At each site, 1 mL of seed inoculum was aseptically transferred into three Erlenmeyer flasks for each medium formulation (*n* = 3, 250 mL flasks each containing 30 mL of medium). Three uninoculated flasks were also prepared for each medium formulation as a medium “blank” (control). Inoculated flasks were incubated at 22 °C, standing, in the dark for a period of 14 days. 

### 2.3. Single-Site Study

For the single-site study, seed inoculum was prepared for each of the three different *A. aculeatus* ex-type strains (NRRL 20623, ATCC 16872, and BCRC 32190) and 1 mL of inoculum was aseptically transferred into nine Erlenmeyer flasks for each medium formulation (*n* = 9, 250 mL flasks each containing 30 mL of medium). A total of five uninoculated flasks (of each fermentation medium) were also carried through the fermentation conditions as medium “blanks” to be used as controls in the data analysis. Inoculated flasks were incubated at 22 °C, standing, in the dark for a period of 14 days. Two weeks later, a second round of seed inoculum of *A. aculeatus* strain BCRC 32190 was prepared and used to inoculate nine replicate 250 mL flasks (*n* = 9, for each of the five fermentation media) that were incubated for 14 days (along with medium “blanks”) for a data comparison to assess for experimental reproducibility between fermentation conditions. 

### 2.4. Extraction and UPLC-HRMS Analysis

Each fermentation flask was extracted with 30 mL of ethyl acetate for 1 h, shaking gently at 150 rpm. A 12 mL aliquot of the organic phase was then removed and liquid partitioned with an equivalent volume of deionized H_2_O. From the organic phase, a 5 mL aliquot was then transferred into pre-weighed glass vials and then dried under a stream of air or nitrogen. 

Based on determined extract weights, samples were resuspended in methanol to yield a final concentration of 500 µg/mL. Prior to UPLC-HRMS analysis, an aliquot of internal standard (dioctyl phthalate) was added to each sample to be used as a quality control for data preprocessing. UPLC-HRMS analysis was carried out using a Kinetex 1.7 µm C_18_ column (Phenomenex, 50 × 2.1 mm) and an Accela Thermo UPLC with a Thermo Exactive mass spectrometer (ThermoFisher Scientific, Waltham, MA, USA) in ESI+ mode (with a *m/z* 190–2000 mass range).

### 2.5. Data Processing

Data preprocessing was carried out using mzMine2 (Cell Unit, Okinawa Institute of Science and Technology (OIST), Onna, Okinawa, Japan). UPLC-HRMS data files were examined (including media controls and MeOH blanks along with treatment data) to determine a minimum noise level threshold. The Full Width at Half Maximum (FWHM) algorithm was used for mass detection with a noise level cut off of 2.0 E^4^. UPLC-HRMS data were deconvoluted, aligned, isotopes removed and then converted into a data matrix of discriminate variables (based on RT and *m/z*) based on peak area measurements. Peak area was normalized by dividing by the total ion current for each sample. Peak alignment was made using the Join Alignment function (with a *m/z* tolerance of 5.0 ppm and a RT tolerance of 0.1 min with a 20:10 weight for *m/z* vs. RT). A gap-filling algorithm was then applied to fill in gaps in the data matrix with detected variable values that fell below the noise limit threshold (2.0 E^4^) during mass detection (using a *m/z* tolerance of 5 ppm and RT tolerance of 0.05 min). 

The data matrix was then visualized by plotting a heatmap in Excel. Redundant variables associated with medium “blank” controls and MeOH blanks were removed from the data matrix. To further visualize the data structure, the data matrix was uploaded into the R environment and the data was centered (variable fluctuations around the mean were centered to fluctuations around zero) and scaled using pareto scaling (using the square root of the standard deviation as the scaling factor). Hierarchical cluster analysis was then performed on the scaled data matrix using the Euclidean distance to compute the distance matrix and single, complete and UPGMA (Unweighted Pair Group Method with Arithmetic Mean) as the linkage criteria. Both multivariate and univariate statistical analyses were completed with the *muma* package (metabolomics univariate and multivariate analysis; [19]. Univariate statistical analysis was carried out using a decisional tree algorithm where Shapiro Wilk’s test for normality was performed to determine if each variable was normally distributed or not and to decide whether a parametric (Welch’s *t*-test) or a non-parametric test (Wilcoxon-Mann Whitney test) was warranted. The univariate function in *muma* then merged the *p*-values deriving from Welch’s *t*-test and from Wilcoxon-Mann Whitney Test, according to the distribution of each variable (Shapiro Wilk’s *p*-value > 0.05 for normal distribution, Welch’s *t*-test; *p*-value < 0.05, Wilcoxon-Mann Whitney Test); in this way, for each pairwise comparison a file was created with merged *p*-values from the two tests. The univariate function also calculated log fold change increases between pairwise comparisons (between treatments, e.g., 0% control vs. 100% seawater) and carried out an ANOVA analysis for each variable. 

## 3. Results 

### 3.1. Characterization of A. aculeatus Ex-Type Strains

**Figure 1:**
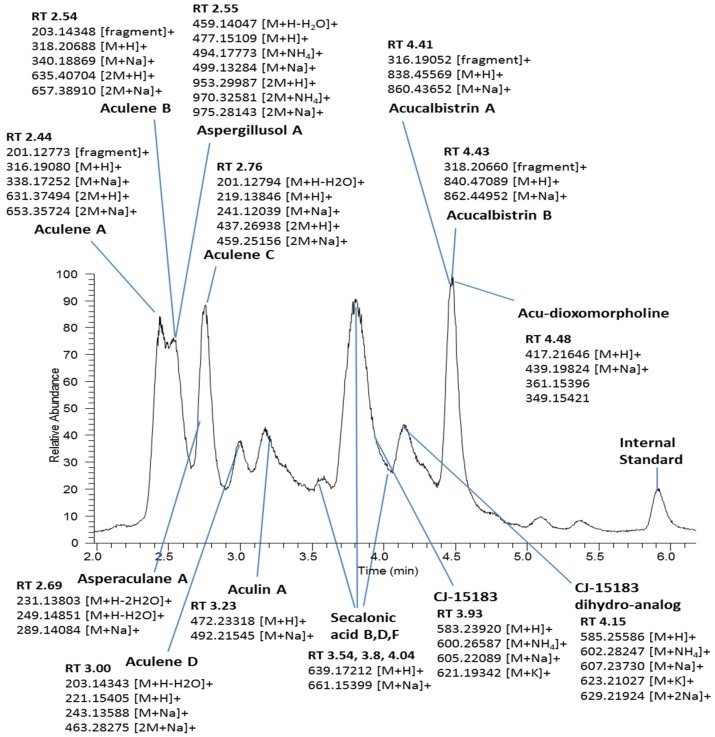
Annotated total ion current chromatogram of *A. aculeatus* ex-type strain ATCC 16872 (0% alt/glycerol control).

Robust growth was observed for each of the three *A. aculeatus* ex-type strains when exposed to increasing concentrations of glycerol and seawater salinity. All standing cultures were visually inspected and no apparent differences in colony morphology were observed between medium controls and treatment conditions. Mycelial mats extended to the walls of the flasks, covering the medium surface, with abundant sporulation. Ethyl acetate extracts of the cultures were chemically profiled using UPLC-HRMS to assess for secondary metabolite differences between culture conditions. A list of known secondary metabolites associated with *A. aculeatus* was compiled from the literature in order to associate pseudomolecular ion signals (variables) with known molecules. Signal annotation was possible for fourteen metabolites (see Figure 1) based on reported metabolite elution order in RP chromatography and associated pseudomolecular ion *m/z* data: aculene A–D, aspergillusol A, asperaculane A, aculin A, acucalbistrin A and B, acu-dioxomorpholine, CJ-15183 (and a novel dihydro-analog), and secalonic acids B, D and F [20,21,22,23,24,25,26]. Assignment of the [M + H]^+^ pseudomolecular ion was supported by observed adduct ions and known fragment ions sharing exact chromatographic peak shapes and retention times using single ion monitoring. Pseudomolecular ions associated with four additional metabolites (asperaculane B, okaramines A, H and J) were also observed; however, abundance levels for these ions fell below the threshold cut-off for peak assignment during data preprocessing and therefore were not included in the metabolomic analysis of the data set. 

### 3.2. Multi-Site Study: Summary

**Figure 2:**
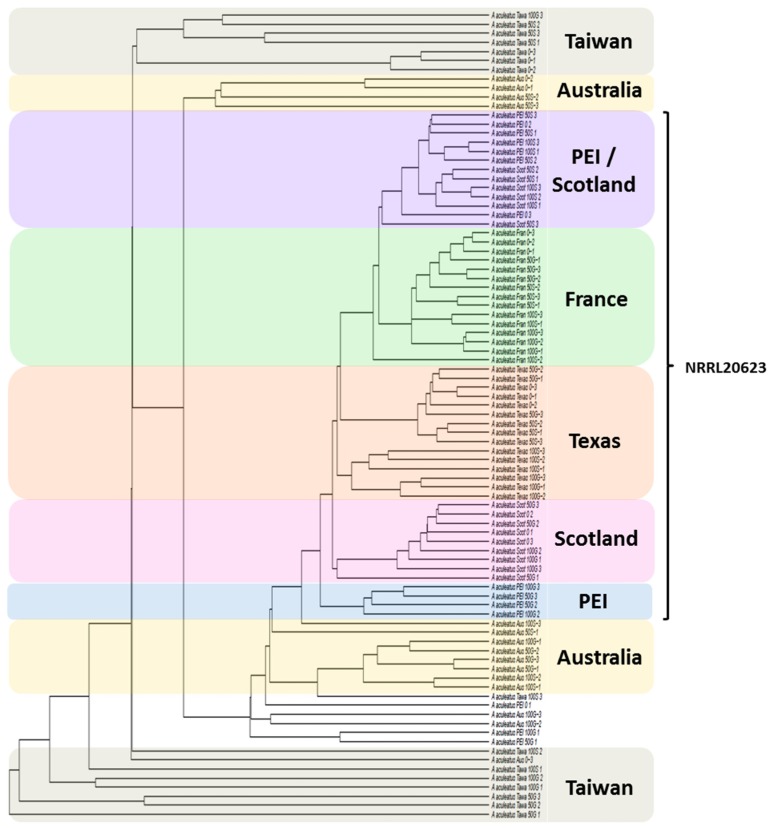
Single linkage cluster analysis of the complete *A. aculeatus* data set (all sites, all treatments).

**Figure 3:**
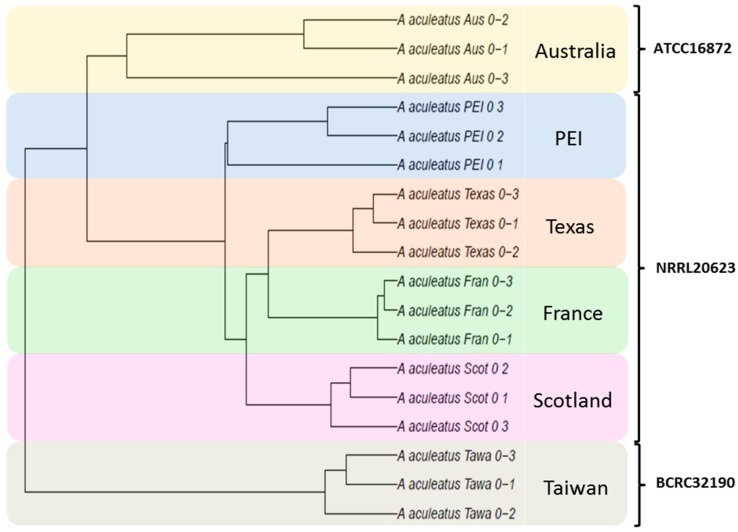
Single linkage cluster analysis of the *A. aculeatus* 0% osmolite control treatment (all sites, single treatment).

A “first pass” cluster analysis was performed on the multi-site data set (using both single linkage and complete linkage criteria) to inspect the “structure” of the data. Looking at the overall data structure, obvious structure relating to a consistent secondary metabolism response to seawater or glycerol treatments was absent (Figure 2). Interestingly, the data structure modeled differences between sites as evident from the formation of site-specific clades (whereas if the data structure were to indicate differences between treatments, clades would consist of treatments representing multiple sites). To account for contaminant ions (MS contaminants) commonly detected by mass spectrometry (pseudomolecular ions associated with plasticizers and detergents), the variable peak list was cross-referenced against a published list of common MS contaminants [27]. Site-specific differences in the presence of MS contaminants were observed (Figure S1) and these variables were subsequently removed from the data set. However, following removal of the MS contaminant variables, the site-specific clustering observed in Figure 2 remained. To reduce the complexity of the data, cluster analysis was performed using data sets consisting of only one treatment. Cluster analysis results were consistent between each of the individual treatment data sets. Triplicates grouped together into individual clades demonstrating reproducibility between replicates and that the observed differences in metabolism were site based (e.g., Figure 3: dendrogram of the 0% osmolite control samples; see Figure S2 for representative UPLC-HRMS chromatographic profiles). Distinct clades were also observed representing the culture collection from which the *A. aculeatus* ex-type strain was obtained indicating that differences in metabolite expression could not be contributed to site specific differences alone, but also relate to the source of the ex-type strain (ATCC 16872 was obtained by the Australian group, BCRC 32190 was obtained by the Taiwanese group, and NRRL 20623 was shared between the Scottish, Canadian, French and American groups). Follow-up correspondence between the six collaborating research groups led to the discovery that slight deviations in the experimental protocol were implemented at the different research sites (e.g., flasks were covered by cotton bungs, milk filters, and screw caps that would affect air exchange in the culture flasks; and different sources of natural seawater were used). 

### 3.3. Single-Site Study: Summary

**Figure 4:**
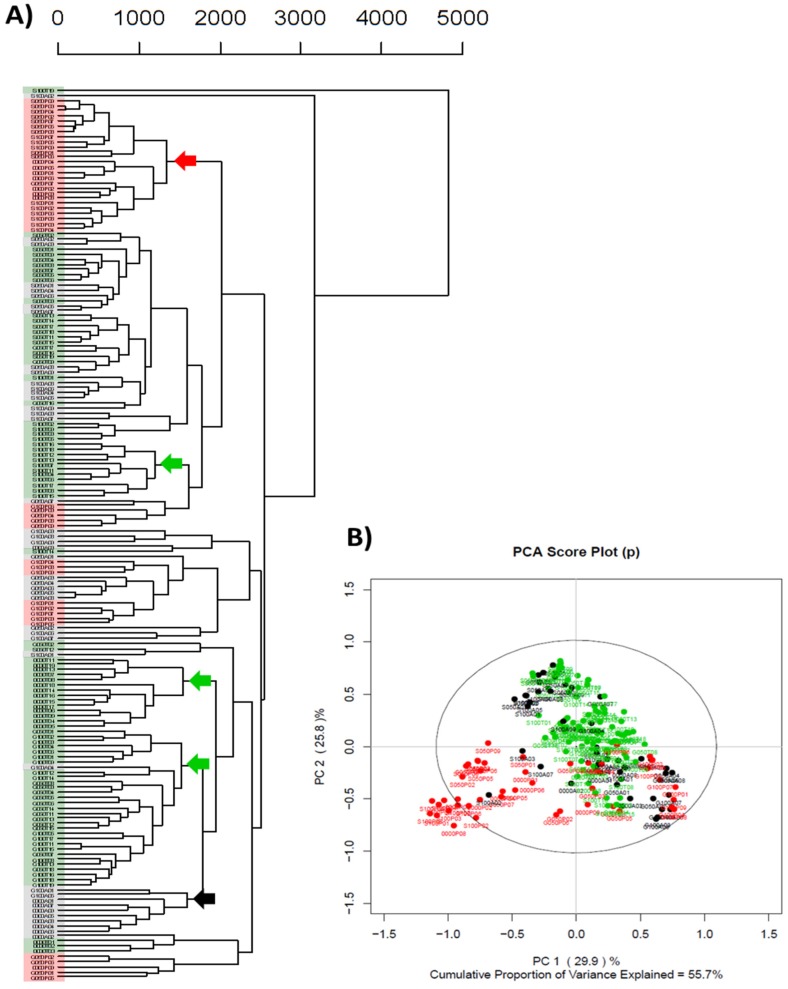
(**A**) Cluster analysis dendrogram of complete data set (pareto scaling, Eucledian distance function, UPGMA linkage criterion). Arrows indicate relevant nodes and coloration denote ex-type strain (black for ATCC 16872, green for BCRC 32190, red for NRRL 20623); (**B**) Principal component analysis score plot for PC1 vs. PC2 (PCA of complete data set).

To compensate for site-to-site variability, the experiment was repeated at a single site and this time nine rather than three replicates were used with each of the three different *A. aculeatus* ex-type strains (ATCC 16872, BCRC 32190, and NRRL 20623). To visualize associated relationships between ex-type strains and culture conditions, hierarchical cluster analysis was performed on the single-site study data set and the resulting dendrogram using the pareto scaling is presented in Figure 4A. In Figure 4, samples were highlighted by color to assess the effect that strain differences had upon the data model: green representing BCRC 32190, red representing NRRL 20623, and black representing ATCC 16872. Strain associated sample coloration within the dendrogram highlighted distinct clades containing samples of the same strain associated with different treatments (clade nodes are highlighted by arrows colored with respect to sample strain, Figure 4A). However several clades of the dendrogram consisted of samples originating from different stains sharing the same fermentation treatments, indicating that data structure alone cannot be explained by strain differences. Principal component analysis was carried out on the pareto scaled data matrix (Figure 4B). The first two principal components explained a combined 57% of the variance in the data model (with approximately 30% of the variance explained by PC1 and 26% of the variance explained by PC2). NRRL 20623 samples associated with different treatments (0% seawater/glycerol control, 50% and 100% seawater) separated from the remaining samples along PC1 indicating differences in secondary metabolite expression of these samples compared to the ATCC 16872 and BCRC 32190 ex-type strains. The PCA data supported the observed formation of a distinct clade within the cluster analysis (red arrow in Figure 4A), confirming that some of the variance in the data model can be explained by differences in secondary metabolite production between the ex-type strains. The grouping of the remaining ATCC 16872 samples (50% glycerol and 100% glycerol) with similar treatment conditions for the other two ex-type strains in the PCA (similar to the cluster analysis results), reinforced the conclusion that the variance in the data model could not be explicitly attributed to differences in secondary metabolite production between the three ex-type strains; rather media composition (treatment) also influenced secondary metabolite production. 

**Figure 5:**
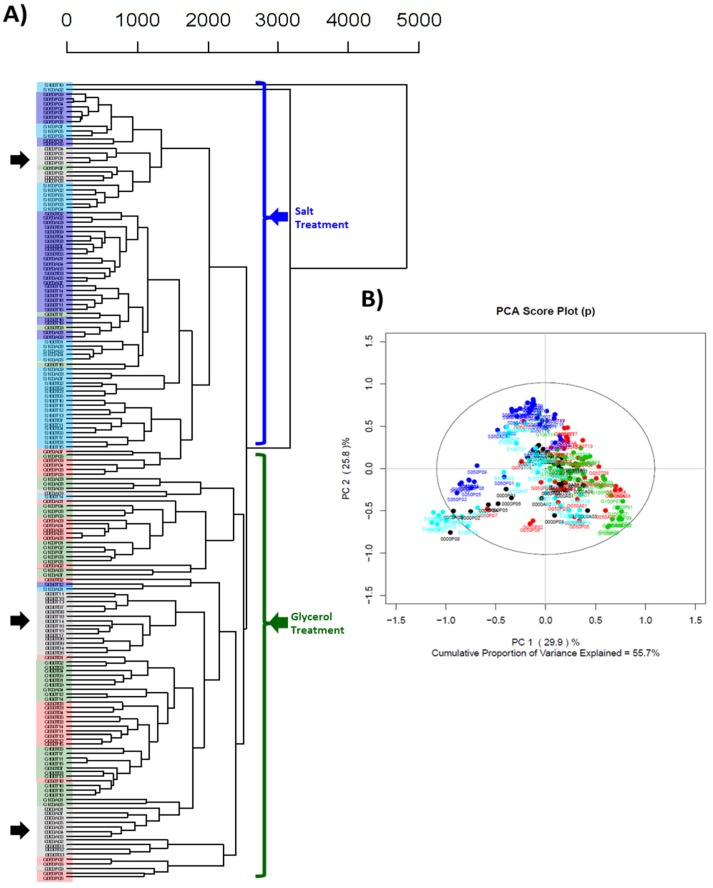
(**A**) Cluster analysis dendrogram of complete data set (pareto scaling, Euclidian distance function, UPGMA linkage criterion). Black arrows indicate clustering of ex-type 0% control treatments. Coloration highlights denote treatment (black: 0% control; red: 50% glycerol; green: 100% glycerol; dark blue: 50% salt; light blue: 100% salt); (**B**) Principal component analysis score plot for PC1 vs. PC2 (PCA of complete data set).

To visualize the effect that medium composition played upon the single-site hierarchical cluster analysis result in Figure 4, samples were colored based on treatment: black/grey for 0% control (0% salt/glycerol), red for 50% glycerol, green for 100% glycerol, dark blue for 50% seawater, and light blue for 100% seawater (as depicted in Figure 5). Two distinct clades based on seawater vs. glycerol treatment were observed; however, 0% control samples also formed individual subclades based on ex-type strain (black arrows in Figure 5). Within the large salt and glycerol treatment clades, samples intermingled between gradations of seawater or glycerol (i.e., samples within the salt clade do not group into uniform subclades based on 50% salt and 100% salt) due to the influence of secondary metabolite production differences between ex-type strains. Therefore, to further explore differences in secondary metabolite expression due to different culture conditions, data sets of the three ex-type strains were independently analyzed. 

**Figure 6:**
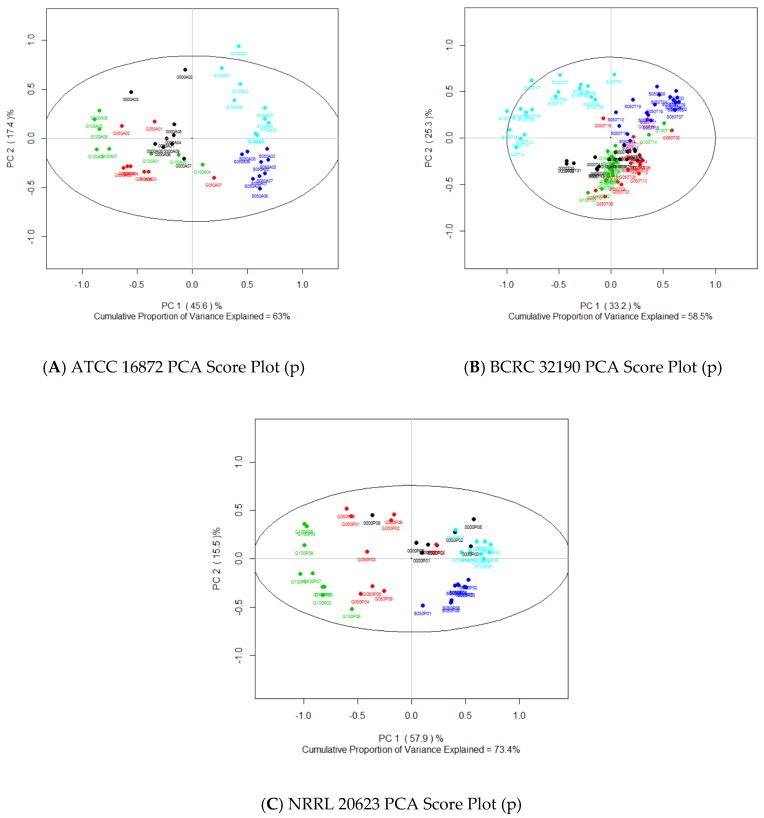
Principal component analysis score plots of PC1 and 2 from ex-type strain data sets: (**A**) ATCC 16872; (**B**) BCRC 32190; (**C**) NRRL 20623.

Principal component analysis of the individual ex-type strains (Figure 6A–C) yielded models with a greater cumulative explained variance for PC1 and 2 (63% for ATCC 16872, 73.4% for NRRL 20623, and 58.5% for BCRC 32190) compared to the previous PCA generated from the combined ex-type strain dataset (Figure 4B). Similar trends were observed in PCA score plots when comparing ex-type strains ATCC 16872 and NRRL 20623 (Figure 6A,C), where salt treatment samples had a positive directional movement along PC1 and glycerol treatments a negative directional movement. In both PCA models of ex-type strains ATCC 16872 and NRRL 20623, 50% salt and 100% salt treatments separate from each other along PC2. In comparison, in the PCA of the BCRC 32190 samples (Figure 6B) both glycerol treatments group together with those of the 0% control samples, with no movement along PC1 and only slight negative movement along PC2. However, saline treatment samples separate from the other treatments (in this case with positive movement along PC2) and from each other (due to negative movement along PC1 for 100% salt and positive movement along PC1 for 50% salt).

### 3.4. Single-Site Study: Effects of Osmolite Concentration upon Secondary Metabolism

**Figure 7:**
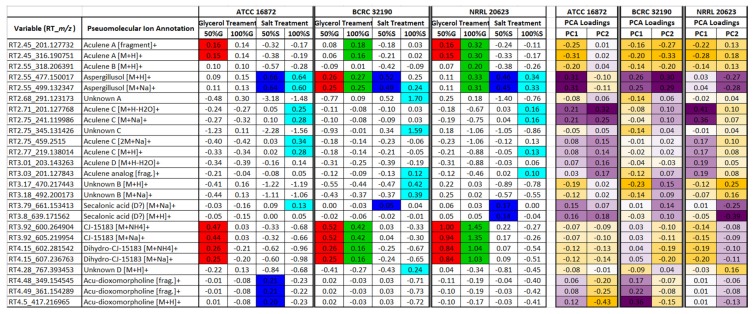
Summary of relevant variables having significant increases in treatment vs. control pairwise comparisons for all three *A. aculeatus* ex-type strains when cultured at a single site (single site comparison). Highlighted cells represent statistically significant (*p*-value < 0.05) log fold increases in peak area (red: 50% glycerol; green: 100% glycerol; dark blue: 50% salt; light blue: 100% salt). Corresponding PCA loading values for PC1 and PC2 associated with modeling of individual ex-type strain data sets (purple represents positive and yellow represents negative loadings, color intensity based on greatest loading value per PC).

Univariate data analyses were carried out to compare the differences in metabolite expression both between treatments for a given ex-type strain as well as between ex-type strains. Significant variables (*p*-value < 0.05) in pairwise comparisons between treatment vs. 0% control for individual ex-type strains were determined and associated log fold changes were calculated (see Table S1). A short list of variables associated with significant fold increases in treatment samples vs. control samples were compiled to reduce the dimensionality of the data set and highlight those variables that contributed the most to the variance in the data model. Significant variables associated with relevant PCA loadings values contributing the most to the variance in the data model, driving sample separation along PC1 and PC2 (based on treatment), were tabulated for each of the three ex-type strains (Figure 7; purple highlights indicate positive loadings values and yellow indicate negative loadings). Trends of significant increased metabolite expression were observed; with particular metabolites have an association with an increase in osmotic potential (red and green highlights; 50% glycerol and 100% glycerol respectively) while others are associated with an increase in salinity (dark and light blue highlights; 50% salt and 100% salt respectively). 

**Scheme 1:**
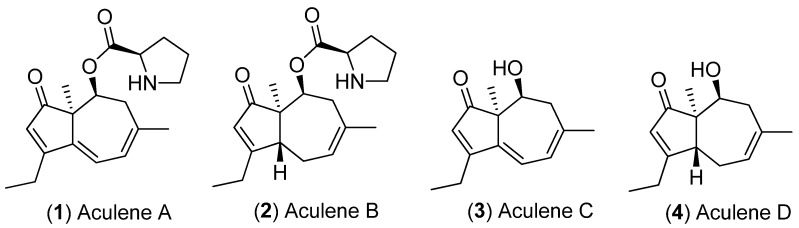
Structures of aculenes A–D.

Aculene A (**1**) and B (**2**) (Scheme 1) both increased in abundance in response to increased glycerol concentrations and decreased when exposed to an increase in salinity (Figures S3, S5 and S6). Pseudomolecular ions associated with these metabolites had high loading values associated with the separation of osmotic stress treatment samples away from saline treatment samples in PCA models for all three ex-type strains (Figure 6A–C and Figure 7). The opposite however was observed for aculene C (**3**) (a presumed biosynthetic precursor to **1** and **2**) and a second metabolite (sharing a characteristic fragment ion *m/z* 201.1278 for the aculenes; Figures S3, S5 and S6), which exhibited significant increases in 100% seawater salt treatments for ex-type strains ATCC 16872 and NRRL 20623 and had high loadings values associated with the separation of 100% seawater treatment samples in the PCA models (Figure 6A,B and Figure 7). Decrease in aculene A (**1**) and B (**2**) production in saline conditions may be related to the increase in aculene C (**3**) (which was more pronounced in the ATCC 16872 and NRRL 20623 ex-type strains).

**Scheme 2:**
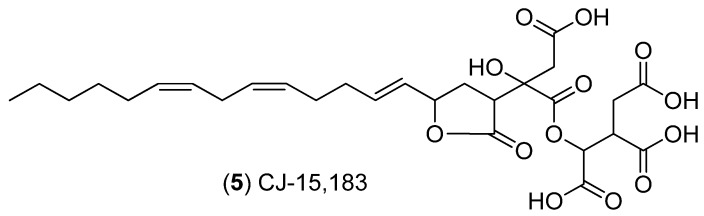
Structure of CJ-15,183.

CJ-15,183 (**5**) (Scheme 2) and an unknown dihydro-CJ-15,183 analog were also observed to increase in conditions of increased osmotic potential. Production differed slightly between ex-type strains in response to osmotic stress. For ex-type strain ATCC 16872, a significant increase in production was observed in 50% glycerol in comparison with observed levels of production at 100% glycerol and in the 0% control (Figure 7 and Figures S3–S12). Both ex-type strains BCRC 32190 and NRRL 20623 demonstrated a significant increase in production under osmotic stress compared to controls for both 50% and 100% glycerol treatments. However for BCRC 32190, production was found to decrease with 50% glycerol and 100% glycerol treatments (similar to ATCC 16872), while production by NRRL 20623 was found to increase significantly with 50% glycerol and increase further with 100% glycerol treatment (Figure 7 and Figures S3–S12). 

**Scheme 3:**
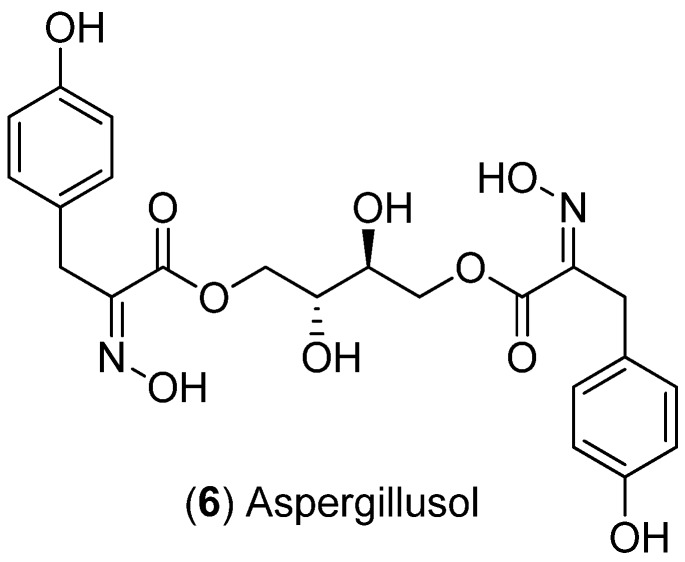
Structure of aspergillusol.

The metabolite aspergillusol (**6**) (Scheme 3) was significantly increased under saline conditions from all three ex-type strains. Significant increase in production of **6** was more pronounced in 50% and 100% seawater treatments compared to glycerol treatments for ex-type strain ATCC 16872 (Figure 7, Figures S3 and S10). For this reason, the [M + H]^+^ and [M + Na]^+^ pseudomolecular ions for aspergillusol had the highest loading values associated with the separation of saline treatments away from the osmotic stress and control treatments in the ATCC 16872 PCA data model (Figure 6A and Figure 7). Significant increases in **6** production compared to controls for ex-type strain BCRC 32190 were observed for both osmotic stress treatments (50% glycerol and 100% glycerol), albeit greater production was observed in saline treatments (especially in 50% seawater; Figure 7 and Figure S3). This observation was reflected in the PCA analysis of the BCRC 32190 ex-type strain dataset as both pseudomolecular ions observed for aspergillusol had substantial loadings in the PCA model associated with the separation of saline treatments (specifically 50% seawater; Figure 6B and Figure 7). Aspergillusol production by ex-type strain NRRL 20623 was similar to that of BCRC 32190, with significant increases in production associated with 100% glycerol, 50% and 100% seawater treatments (Figure 7, Figures S3 and S15) and PCA loading values associated with the differentiation of 50% and 100% seawater treatments along PC2 in the NRRL 20623 PCA data model (Figure 6C and Figure 7). 

**Scheme 4:**
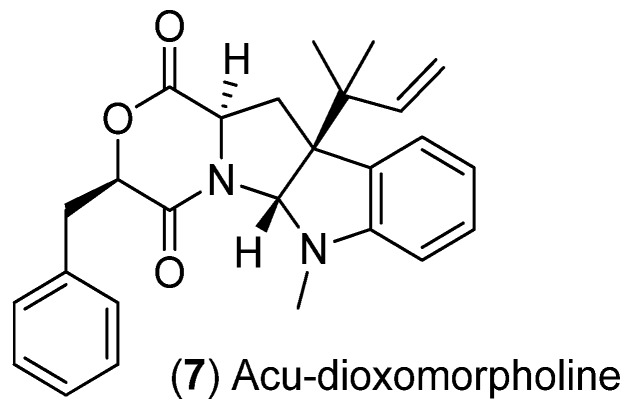
Structure of acu-dioxomorpholine.

Acu-dioxomorpholine (**7**) (Scheme 4) was found to increase significantly only in the 50% seawater treatment with ex-type strain ATCC 16872 (Figure 7 and Figure S3). However significant decreases in production were observed in the 100% seawater treatment for all three ex-type strains. Pseudomolecular ions associated with acu-dioxomorpholine had an important role in the separation of the 50% seawater treatment samples away from the 100% seawater treatment samples along PC2 in the ATCC 16872 ex-type strain PCA data model (due to high negative loading values; Figure 6A and Figure 7). No significant increase or decrease was observed for acu-dioxomorpholine in either of the glycerol treatments compared to the control for each of the three ex-type strains. 

**Scheme 5:**
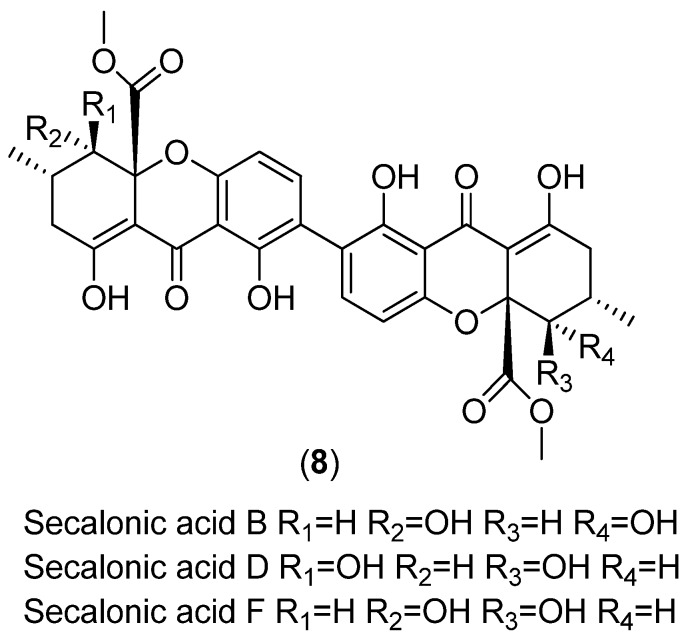
Structures of secalonic acids B, D and F.

A significant increase in secalonic acid (Scheme 5) production (likely secalonic acid D as this has been previously reported as being the more abundant secalonic acid produced by *A. aculeatus* [23]) was observed for ex-type strain ATCC 16872 in the 100% seawater treatment, while the 100% glycerol treatment led to a significant decrease in production in comparison to 0% control samples (Figure 7, Figures S3 and S14). The increase in production observed in the 100% seawater treatment had a moderate effect upon the ATCC 16872 ex-type strain PCA data model causing the separation of saline conditions from glycerol conditions along PC1 (positive loading values) and the separation of 100% seawater from 50% seawater treatment samples along PC2 (Figure 6A and Figure 7). A more pronounced increase in both [M + H]^+^ and [M + Na]^+^ pseudomolecular ions were observed in 50% seawater treatments for ex-type strain NRRL 20623 (Figure 4 and Figure 7). These variables in particular strongly influenced the separation of the 50% seawater treatment samples from the 100% seawater in the NRRL 20623 ex-type PCA data model due to high negative loading values along PC2 (Figure 6C and Figure 7). A slight increase in production of secalonic acid was observed under saline treatment conditions for ex-type strain BCRC 32190; however these increases were not statistically significant.

## 4. Discussion

When a fungal species is formally described, often a living strain is deposited in a recognized public culture collection in order to fix the name to a living strain; this strain is designated as the type strain and ideally, multiple copies are stored in a metabolically inactive state, either in a lyophilized or ultra-cold frozen state. Descendent of the original living tissues obtained from the type strain are designated as ex-type strains and are often distributed to multiple public service collections. Traditionally, genetic resource centers (such as culture collections) support scientific research by providing cultures, including ex-type cultures, to scientists [28]. Proper culture collection curation and handling of strains is essential to preserve ex-type strains that remain representative of the original type specimen. Phenotypic deviations are frequently reported by researchers when investigating ex-type and conspecific strains obtained from various different culture collections [29]. In our case, *A. aculeatus* is a black-spored species (*Aspergillus* section *Nigri*) that is morphologically similar to at least 18 different black-spored species [30]. PCR-fingerprinting is a reliable means of confirming the correct designation of ex-type strains obtained from different culture collections [29]. In our experiment, comparison of DNA sequences (ITS1-5.8s-ITS2 and the D1/D2 variable region of the nLSU rDNA) confirmed that the *A. aculeatus* ex-type strains ATCC 16872, BCRC 32190 and NRRL 20623 were the same species (100% similarity, data not shown). Therefore, differences in the observed secondary metabolite profiles were assumed to be ex-type strain dependent, likely due to somatic changes and degeneration or mutations occurring during successive transfers between periods of long-term storage of the ex-type stock strains.

### 4.1. Site-to-Site Variability

Although a standardized protocol was circulated among the participating laboratories to minimize operational variations, site-specific variations in secondary metabolism were observed (Figure 2; comparison of UPLC-HRMS profiles in Figure S2). During initial data analysis, various pseudomolecular ions representing plasticizers and detergents (“MS contaminants”), were found in all extracts, and the constituents varied between sites. Such contaminants originate from detergents and plasticware used in the laboratory (e.g., sample bottles, vials, pipette tips, pipette bulbs and filter membranes) that differ from lab-to -lab [27]. After removal of known contaminant ions from the data set, site-to-site variation was still observed during data analysis.

Variations in secondary metabolite production likely resulted from both biotic and abiotic factors. Variations in fermentation conditions and nutritional composition affect secondary metabolite expression [31,32]. Even for labs that exchanged the same ex-type strain (NRRL 20623), secondary metabolite production differed sufficiently between sites to form distinct site-specific clades as recognized by hierarchical cluster analysis (Figure 2). Media composition may have played a minimal role because most of the ingredients were defined reagent grade chemicals, and all used the same brand of yeast autolysate (Sigma Aldrich 73145). However, the source of seawater varied locally (and likely varied in dissolved organic and inorganic matter content and possibly pH). The recognition of site-specific clades during hierarchical clustering of the control conditions (where seawater and glycerol were absent; Figure 3) for sites that fermented the same ex-type strain indicated that additional factors influenced on secondary metabolism. Sources of undefined medium ingredients (e.g., yeast and malt extracts), may differ in composition even between lot numbers from the same manufacturer, and these subtle difference have a direct impact on secondary metabolite expression from the same strain [33]. Abiotic factors such as aeration may have also been a factor as sites differed in how the Erlenmeyer flasks were closed (some pressure-tight caps, cellulose stoppers, or cotton). While it is impossible to identify specific factors that caused the differences in secondary metabolite production, these data highlight the subtleties in culture conditions that may influence natural product biosynthesis. 

### 4.2. Differences in Ex-Type Strain Secondary Metabolism

When each of the ex-type strains originating from different culture collections were fermented at a single site, only the fermentation condition (saline and osmotic stress) and ex-type strain were found to contribute to the variation in secondary metabolism. Ex-type strains did not segregate uniformly based on treatment following PCA and cluster analysis (Figure 4). Theoretically, genetically equivalent ex-type strains should express a consistent pattern of secondary metabolites under a given fermentation condition as all originated from the same parent. However, this was not the case in this experiment. For example, metabolites 5′-hydroxyasperentin 8-*O*-methyl ether and alantryleunone increased under osmotic stress for ex-type strains ATCC 16872 and NRRL 20623, while production increased under saline stress for ex-type strain BCRC 32190 (Figure S3). 

During prolonged culturing and transfers, filamentous fungi undergo spontaneous somatic changes, referred to as sectoring. Sectors are regions that commonly form in agar culture that can differ in morphology (e.g., lack sporulation), secondary metabolism, and enzyme production from the parent inoculum. Characteristics of a culture sector may remain stable after subculturing. However, sector formation is often associated with physiological instability and frequently results in changes in secondary metabolite production where gains as well as losses have been reported [34,35,36,37]. Although the production of some secondary metabolites can be correlated with morphological changes, e.g., loss of sporulation [38], degeneration in sporulation is not necessarily associated with a loss in secondary metabolite production [33,36]. Similarly, heavily mutated factory production strains may have aberrant morphology, including loss of hydrophobicity, pigmentation, aerial hyphae, and sporulation [39,40]. 

The *A. aculeatus* ex-type strains used in this study originated from the type strain WB 5094 [41]. As the type strain material has been in the collection at least sixty years, repeated handling of ex-type progeny over time at different locations and with different transfer histories has resulted in three ex-type strains that differ in secondary metabolite expression. The stability of secondary metabolite profiles deteriorate over longer storage periods, which can be minimized (but not entirely prevented) by storage in a metabolically inactive state such as lyophilization or cryostorage [28,35]. 

Annotation of the genome of *A. aculeatus* ex-type strain ATCC 16872 indicates >60 gene clusters encoding non-ribosomal peptides, polyketides, terpenoids, and other secondary metabolites, not all of which have been linked with known molecular structure [18]. However, some of the secondary metabolites that have previously been reported by this strain and for which the gene cluster was present, were not observed in this experiment, e.g., aculeacin [42]. For experimental simplicity (and to minimize variability between labs), the extraction protocol used was not exhaustive and would have favored the extraction of metabolites that were present in the culture broth. Secondary metabolites that accumulate within the mycelium or spores of the fungus would therefore have been less prevalent in the extract and might have been missed in the analysis due to their low abundance in the overall extract composition. Pseudomolecular ions relating to several metabolites such as aculeacin, calibistrins, auerospurones were not observed in culture extracts when resulting chromatograms were examined using single ion monitoring. Pseudomolecular ions for other metabolites such as the okaramines and asperaculane B were observed using single ion monitoring but occurred at ion intensities below the noise cutoff threshold used for data analysis. For other metabolites such as the acucalibistrins and asperaculane A where pseudomolecular ions were detected at an intensity above the noise threshold, metabolite changes between fermentation conditions were insignificant. Only half of the variables in the data model could be linked with secondary metabolites previously reported in the *A. aculeatus* literature; the remaining variables represent a considerable number of secondary metabolites present in the metabolome of *A. aculeatus* that have yet to be structurally confirmed. 

### 4.3. Effect of Increased Salinity/Osmotic Stress on Secondary Metabolism

Media compositions containing either increasing concentrations of glycerol or sea salts create an environment of low water potential and thus provide a stress condition for fungi. Some fungi have evolved cellular mechanisms to cope with environments with low water potential, and for this reason, are considered as osmotolerant. In the marine environment, increased salinity exerts an additional stress due to the presence of relatively high concentrations of sodium and chloride ions, which potentially exert toxic effects on cellular processes [43]. Genome characteristics of halophilic fungi include enrichment in genes related to stress response proteins, ion/metabolite transporters, hydrophobins, and polyol biosynthesis (including the HOG pathway) [6,7,8]. Although several mechanisms such as increased polyol biosynthesis are a common response shared between osmotolerant and halotolerant fungi to low water potential, adaptations involving maintenance and efflux of metal ions are only expressed under saline stress induced by exposure to sea salts. Transcriptome studies have demonstrated differences in expression of both primary and secondary metabolites due to salt stress; however, differential induction of several secondary metabolites were found to occur in seawater fermentations compared to fermentations using NaCl alone, where authors attributed induction to occur as a result of upregulation of metal ion efflux [44]. In our project, upregulation of secondary metabolites was found to differ between osmotic stress (induced by glycerol) and saline stress (induced by sea salts). Specifically, production of the metabolites CJ-15,183, a dihydro-CJ-15,183 derivative and aculenes A and B were found to increase in response to increasing glycerol concentration, while production under saline stress conditions was consistent with that of non-stress conditions (0% seawater/0% glycerol control). Other secondary metabolites such as aspergillusol, secalonic acid D, aculene C and another aculene analog were found to be upregulated in saline conditions. Interestingly, aspergillusol was first described from a marine-derived strain (CRI323-04) of *A. aculeatus* fermented in potato dextrose broth using seawater [22]. At the time, Ingavat and collaborators attributed the discovery of aspergillusol to the marine-derived nature of isolate, as it was believed to be adapted to the marine environment and therefore a unique source of new natural products. Based on our results with a terrestrial strain of *A. aculeatus*, expression of aspergillusol was confirmed to be associated with a halotolerant response to saline conditions involving seawater/sea salts. 

Many fungal genera such as *Aspergillus, Penicillium, Trichoderma* and *Cladosporium* have the genetic capacity for prolific production of secondary metabolites, yet generally do not express their full potential for secondary metabolite biosynthesis when cultured in the laboratory. Historically, secondary metabolites associated with saline stress have been overlooked by natural product discovery programs using terrestrial isolates, as the majority of investigators rarely reported fermentation of terrestrial strains using a salt water medium. However, media with high solute concentrations, especially sugars are frequently used. On the other hand, researchers working on marine-derived strains often deliberately include seawater in the fermentation media. This is evident from the numerous reports of new secondary metabolites discovered from commonly occurring, halotolerant fungal genera that have been designated as “marine-derived” [9]. Our findings support the hypothesis that media amended with sea salts (or glycerol) applies a selective pressure on the secondary metabolic output of a fungus, regardless of whether the isolate was of marine or terrestrial origin. Moreover, upregulated production of particular secondary metabolites differs depending on the selective pressure applied (increasing glycerol concentrations vs. increasing seawater salinity). Implications of this discovery suggest that deliberate manipulation of halotolerant fungal strains by additions of solutes, e.g., seawater, salts, or other osmolites, to fermentation media will amplify the kinds of secondary metabolites discovered from these species. Importantly, our findings also highlight the value of multi-investigator, multi-site studies to rigorously assess the effects of the manipulation of culture conditions on natural product production in fungi. It also documents the effect of unforeseen variables on the metabolome extracted from a fungal fermentation, e.g., plasticizers present in common lab plastic-ware, sources of seawater, and degree of culture aeration. 

## 5. Conclusions

This exercise yielded notable conclusions. Firstly, the *A. aculeatus* ex-type strain obtained from different culture collections produced different secondary metabolite expression profiles when fermented at different sites despite using the same fermentation protocols. Secondly, the secondary metabolite profile of *A. aculeatus* fermentations was demonstrated to be responsive to osmotic stress, whether induced by glycerol or seawater. Thirdly, *A. aculeatus* ex-type strains obtained from different culture collections produced different secondary metabolite expression profiles when fermented at the same site using identical fermentation protocols. 

## Supplementary Materials

The following are available online at www.mdpi.com/1660-3397/15/8/254/s1.
