# Supplementary material for: Does Osmotic Stress Affect Natural Product Expression in Fungi?
Source: Mar Drugs. 2017 Aug 13;15(8):254. doi: 10.3390/md15080254 (PMC5577608; doi:10.3390/md15080254)

SUPPLEMENTARY INFORMATION

Does osmotic stress affect natural product expression in fungi?

Overy DP[1,2,3], Correa H[1,4], Roullier C[6], Chi W-C[7,8], Pang K-L[8], Rateb ME[9,10], Ebel R[9], Shang Z[11], Capon RJ[11], Bills GF[12], Kerr RG[1,4,5]

Figure S1: Cluster analysis of pseudomolecular ions associated with known MS contaminants (i.e. plasticizers and detergents) originating from detergents and plastic ware commonly used in the laboratory.

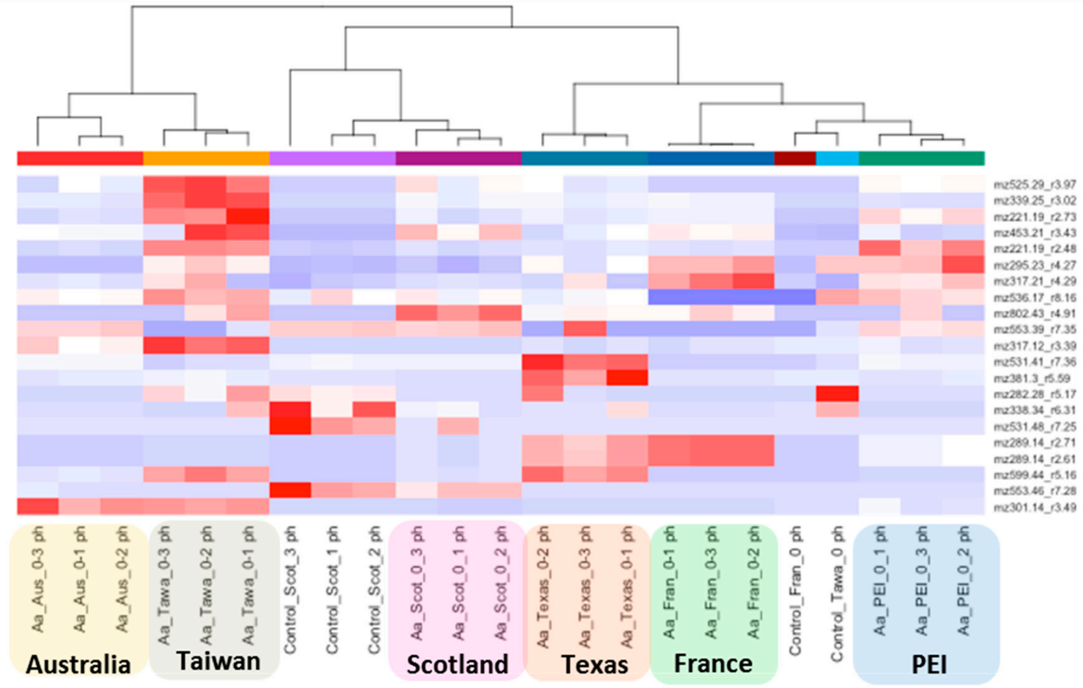

Table S1: Significant variable fold increases from pairwise comparisons (treatment vs 0% control) for each of the three individual ex-type strain datasets (**NOTE: See accompanying excel file to be used as Supplementary Table 1**).

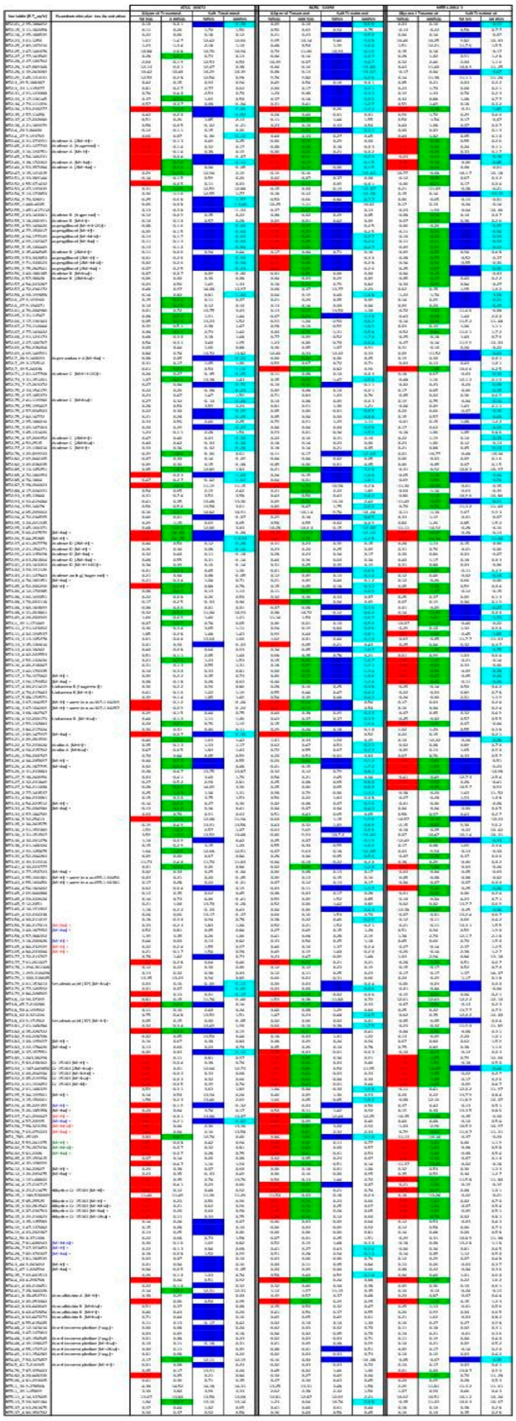

Figure S2: Relevant pseudomolecular ion annotations and associated variable box plots for aculene A (ex-type strain ATCC 16872).

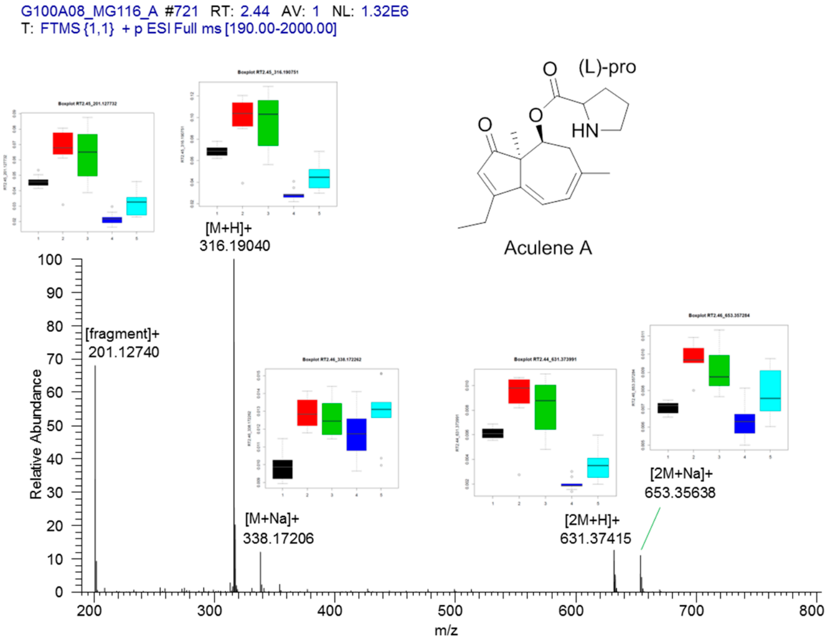

Figure S3: Relevant pseudomolecular ion annotations and associated variable box plots for aculene B (ex-type strain ATCC 16872).

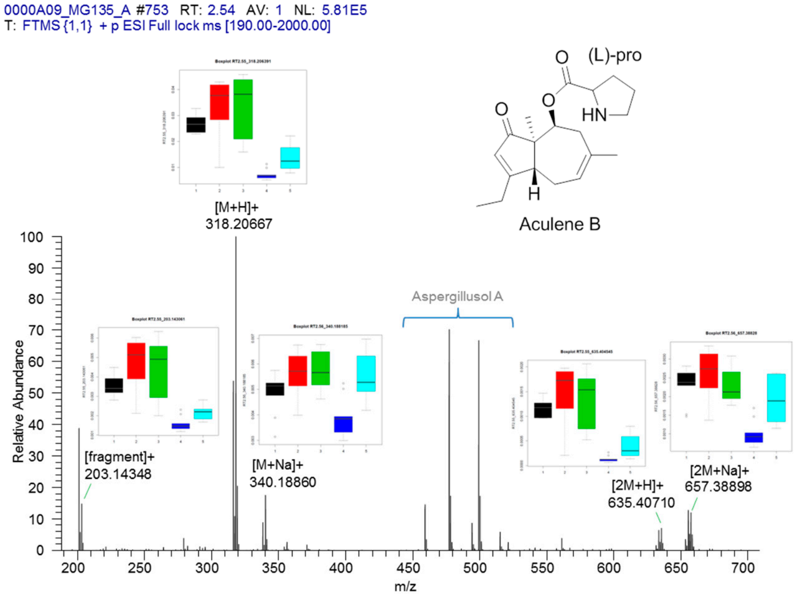

Figure S4: Relevant pseudomolecular ion annotations and associated variable box plots for aculene C (ex-type strain ATCC 16872).

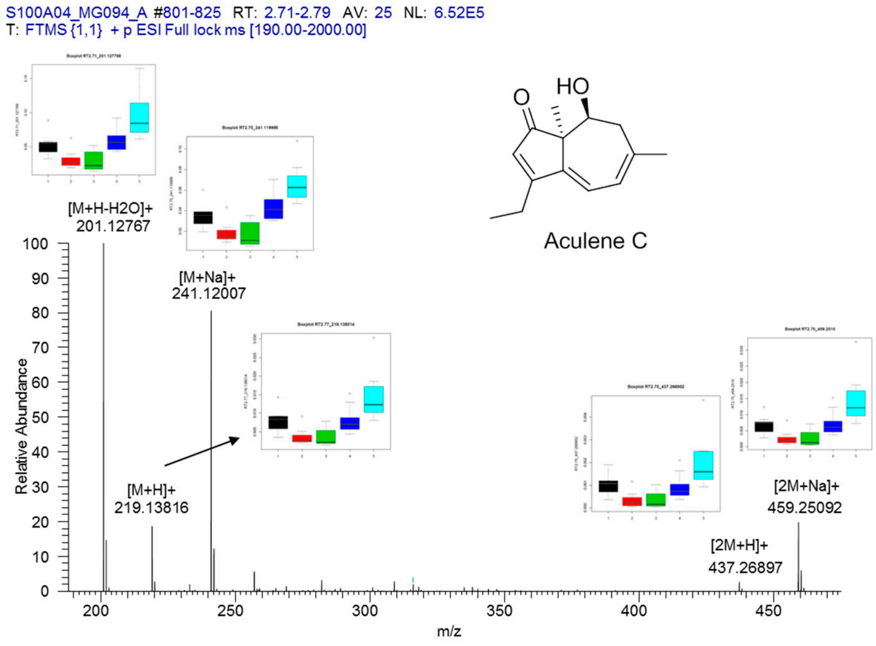

Figure S5: Relevant pseudomolecular ion annotations and associated variable box plots for aculene D (ex-type strain ATCC 16872).

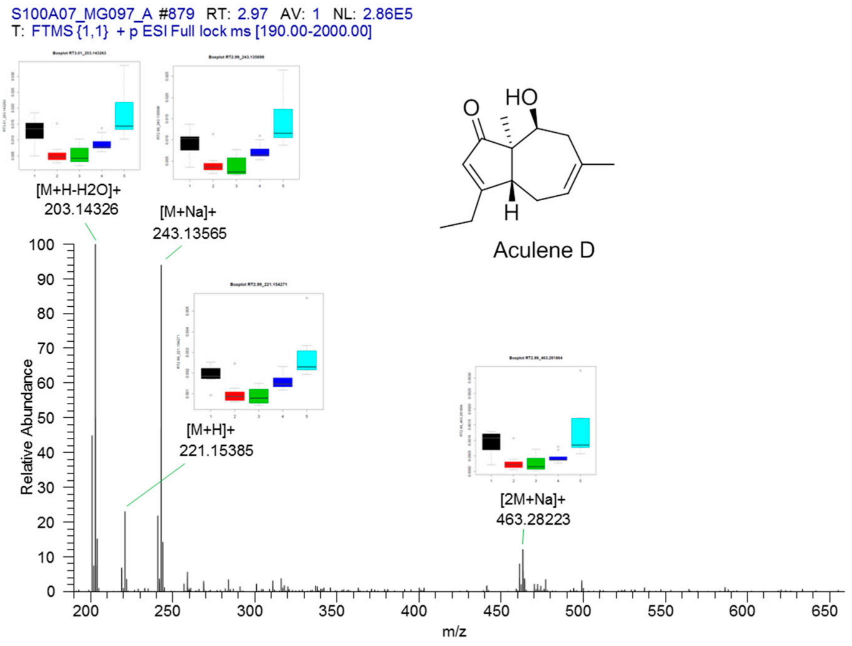

Figure S6: Relevant pseudomolecular ion annotations and associated variable box plots for asperaculane (ex-type strain NRRL 20623).

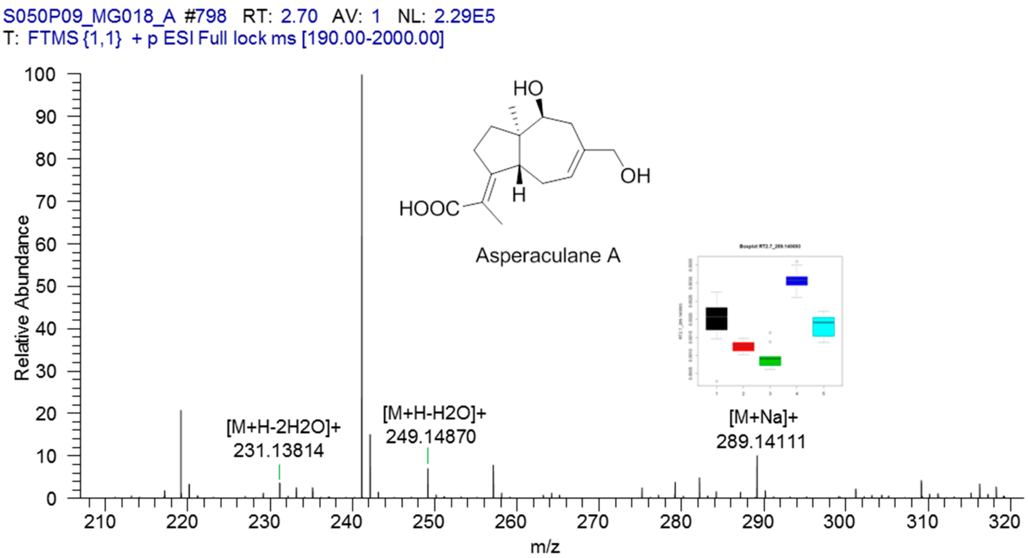

Figure S7: Relevant pseudomolecular ion annotations and associated variable box plots for aspergillusol (ex-type strain ATCC 16872).

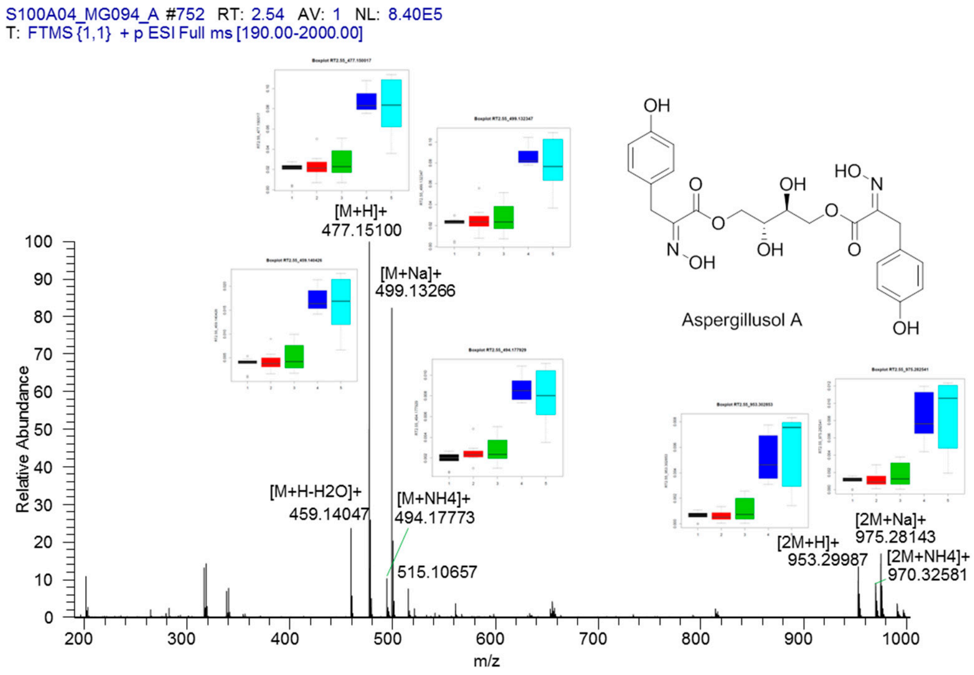

Figure S8: Relevant pseudomolecular ion annotations and associated variable box plots for aculin A (ex-type strain BCRC 32190).

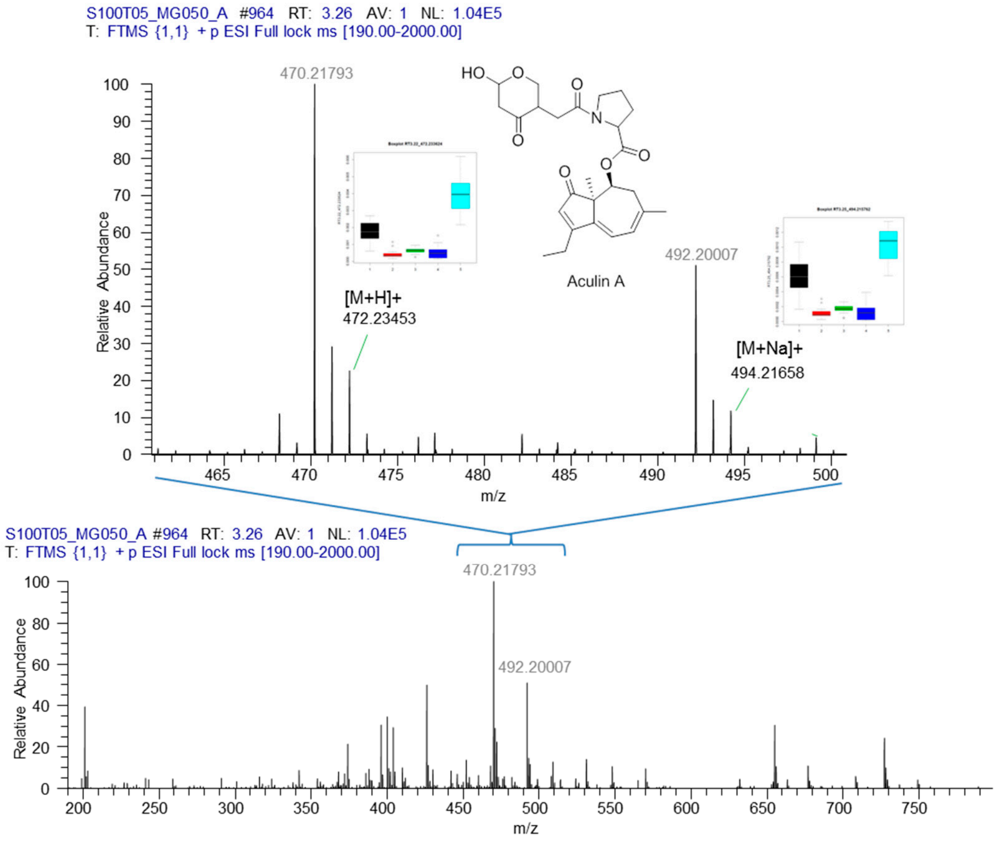

Figure S9: Relevant pseudomolecular ion annotations and associated variable box plots for CJ-15,183 (ex-type strain NRRL 20623).

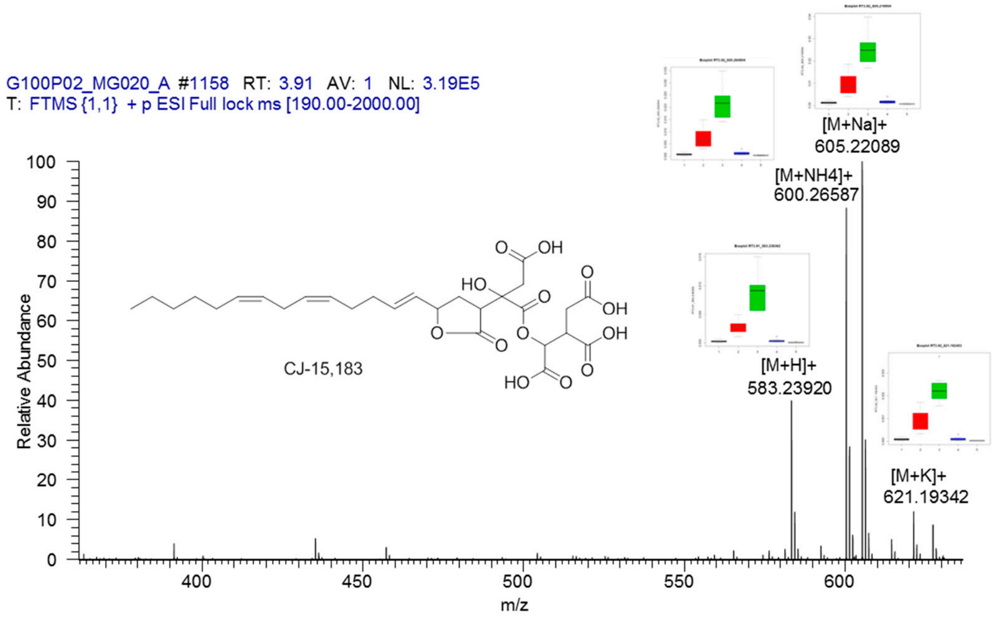

Figure S10: Relevant pseudomolecular ion annotations and associated variable box plots for CJ-15,183 (ex-type strain NRRL 20623).

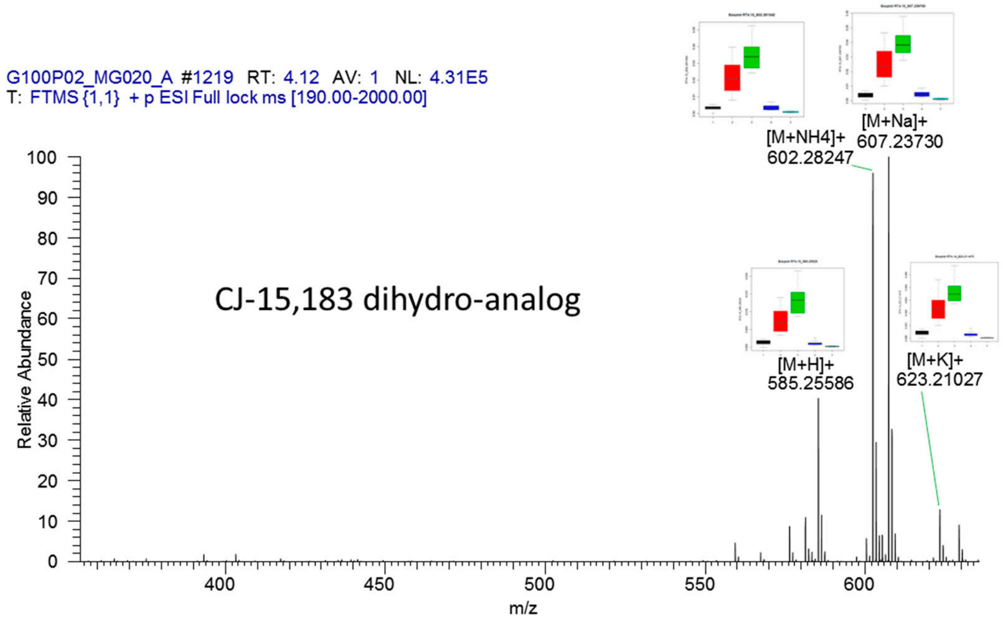

Figure S11: Relevant pseudomolecular ion annotations and associated variable box plots for secalonic acid (most likely secalonic acid D) (ex-type strain ATCC 16872).

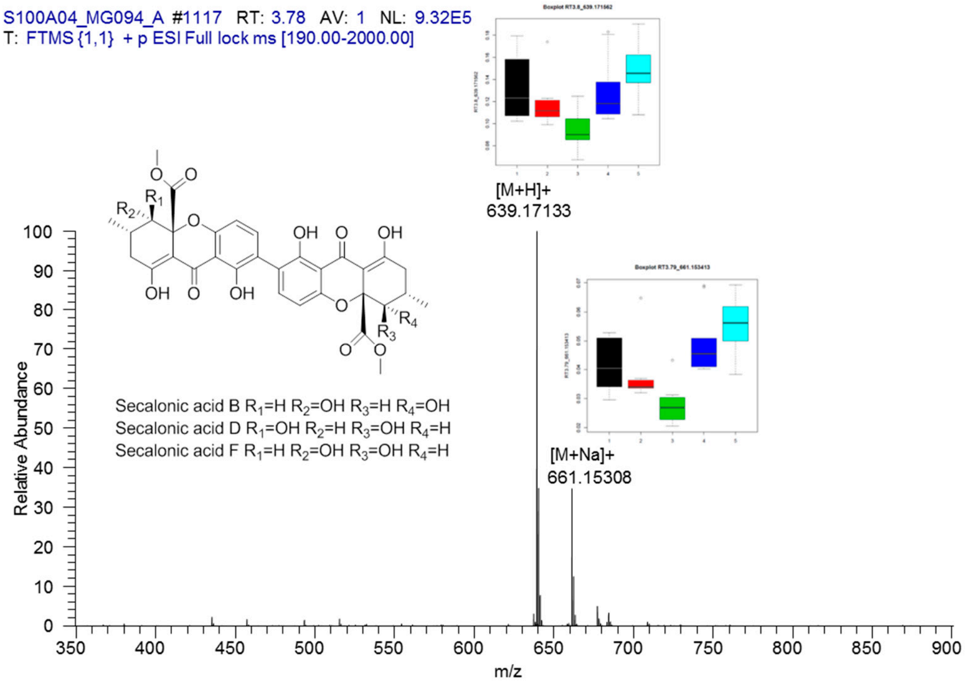

Figure S12: Relevant pseudomolecular ion annotations and associated variable box plots for acu-dioxomorpholine (ex-type strain ATCC 16872).

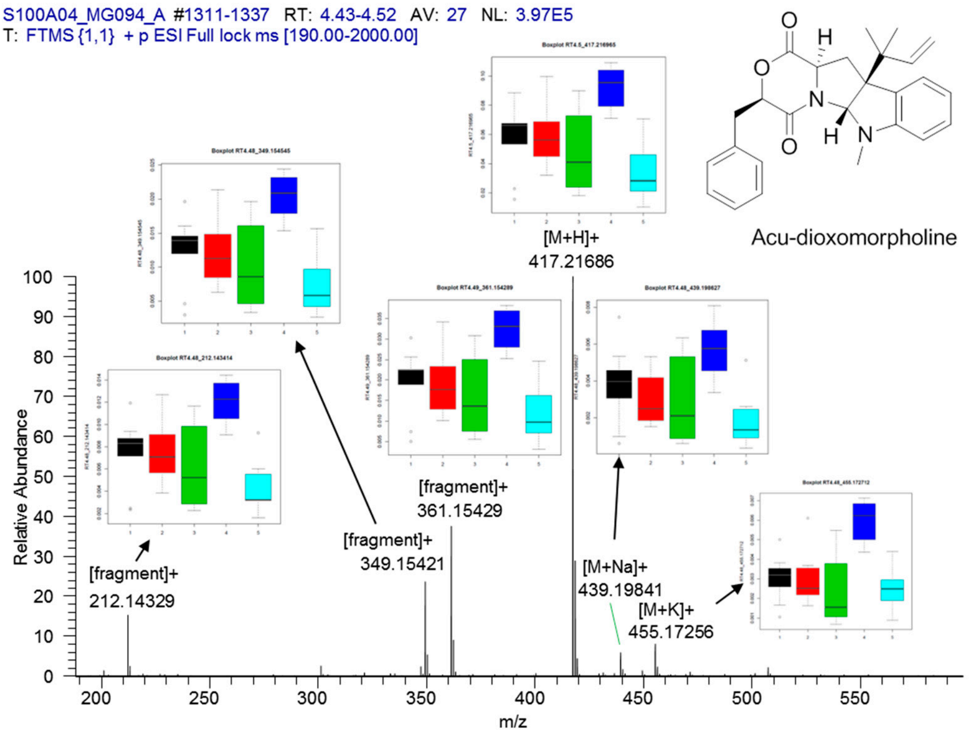

Supplement: Supplementary file 1 [file marinedrugs-15-00254-s001.pdf]
